# SARS-CoV-2-Spike Antibody and T-Cell Responses Elicited by a Homologous Third mRNA COVID-19 Dose in Hemodialysis and Kidney Transplant Recipients

**DOI:** 10.3390/microorganisms10112275

**Published:** 2022-11-16

**Authors:** Nayara Panizo, Estela Giménez, Eliseo Albert, Joao Zulaica, Alicia Rodríguez-Moreno, Luciana Rusu, Elena Giménez-Civera, Maria Jesús Puchades, Luis D’Marco, Lorena Gandía-Salmerón, Ignacio Torres, Asunción Sancho, Eva Gavela, Miguel Gonzalez-Rico, Marco Montomoli, Carmen Maria Perez-Baylach, Begoña Bonilla, Camila Solano, Mª Fernanda Alvarado, Isidro Torregrosa, Boris Gonzales-Candia, María Jesús Alcaraz, Ron Geller, José Luis Górriz, David Navarro

**Affiliations:** 1 Nephrology Service, Hospital Clínico Universitario de Valencia, INCLIVA Health Research Institutue, 46010 Valencia, Spain; 2 Microbiology Service, Hospital Clínico Universitario de Valencia, INCLIVA Health Research Institute, 46010 Valencia, Spain; 3 Institute for Integrative Systems Biology (I2SysBio), Universitat de Valencia-CSIC, 46980 Valencia, Spain; 4 Universidad Cardenal Herrera-CEU Medicine Department, CEU Universities, 46115 Valencia, Spain; 5 Nephrology Service, Transplant Unit, Hospital Universitario Dr. Peset, 46017 Valencia, Spain; 6 B BraumAvitum Hemodialysis Centres Valnefron Valencia and Massamagrell, 46021 Valencia, Spain; 7 Department of Medicine, School of Medicine, University of Valencia, 46010 Valencia, Spain; 8 Department of Microbiology, School of Medicine, University of Valencia, 46010 Valencia, Spain

**Keywords:** mRNA COVID-19 vaccine, SARS-CoV-2-S antibodies, SARS-CoV-2-S T cells, neutralizing antibodies, hemodialysis, kidney transplantation

## Abstract

The effect of a third vaccine dose (3D) of homologous mRNA vaccine on blood levels of SARS-CoV-2-receptor binding domain (RBD)-total antibodies was assessed in 40 hemodialysis patients (HD) and 21 kidney transplant recipients (KTR) at a median of 46 days after 3D. Anti-RBD antibodies were detected in 39/40 HD and 19/21 KTR. Overall, 3D boosted anti-RBD antibody levels (median: 58-fold increase). Neutralizing antibodies (NtAb) against the Wuhan-Hu-1, Delta, and Omicron variants were detected in 14, 13, and 11 out of 14 HD patients, and in 5, 5, and 4 out of 8 KTR patients, respectively. The median fold increase in NtAb titers in HD patients was 77, 28, and 5 and 56, 37, and 9 in KTR patients for each respective variant. SARS-CoV-2-S S-IFN-γ-producing CD8^+^ and CD4^+^ T-cell responses were detected in the majority of HD (35 and 36/37, respectively) and all KTR (16/16) patients at 3D. Overall, the administration of 3D boosted T-cell levels in both population groups. In conclusion, a homologous mRNA COVID-19 vaccine 3D exerts a booster effect on anti-RBD antibodies, NtAb binding to Wuhan-Hu-1, Delta, and Omicron variants, and SARS-CoV-2-S-IFN-γ-producing T cells in both HD and KTR patients. The magnitude of the effect was more marked in HD than KTR patients.

## 1. Introduction

Patients on maintenance hemodialysis (HD) and kidney transplant recipients (KTR) are at increased risk for a severe COVID-19 clinical course [1,2]. HD and KTR patients have been prioritized for access to homologous or heterologous vaccine booster shots due to their particular vulnerability to SARS-CoV-2 infection and because full vaccination schedules are often insufficient to elicit protective levels of anti-Spike (S) antibodies. These patients also exhibit accelerated waning of post-vaccination antibody levels, a concerning fact given the current dominance of the Omicron variant, which partially escapes neutralization by antibodies elicited by mRNA COVID-19 vaccines. This strategy seems particularly necessary in KTR, given that a non-negligible percentage of patients fail to mount detectable adaptive immune responses after two mRNA COVID-19 vaccine doses [2]. The RECOVAC study showed similar vaccination effectiveness between 159 HD patients and 191 controls in terms of seroconversion, while KTR (288) patients showed a worse response. Neither presented safety issues; indeed, systemic vaccination-related adverse events seemed less remarkable in these groups than in controls [3]. Similar results were obtained in the SENCOVAC study in 301 HD patients and 283 KTR patients. Again, no adverse events were registered [4].

To our knowledge, there are not yet any published articles focused on clinical effectiveness. Most studies to date assessing SARS-CoV-2-Spike (S)-immune responses following a third mRNA COVID vaccine dose (3D) in HD and KTR patients are centered on anti-SARS-CoV-2-S-antibody responses, and to a lesser extent, on NtAb, targeting the ancestral Wuhan-Hu-1, Alpha, or Delta variants. These studies showed a booster effect of variable size in the two group populations. Among KTR who failed to develop detectable antibodies after the second vaccine dose, the percentage of responders after 3D varied substantially across the studies [5,6,7,8,9,10,11,12,13,14,15,16,17,18,19,20,21,22,23]. Fewer studies have assessed the impact of the receipt of 3D on SARS-CoV-2-S-targeted functional T-cell responses [5,18,19,20,21,22], which crucially contribute to providing protection against SARS-CoV-2 infection. Studies including KTR also showed a widely varying booster effect [18,19,20,21]. 

Here, we comprehensively investigated the effect of a homologous mRNA vaccine 3D on blood levels of SARS-CoV-2-receptor binding domain (RBD)-total antibodies, NtAb binding to the SARS-CoV-2 variants of concern Delta and Omicron (the latter one with almost worldwide predominance at the time of publication), and S-reactive IFN- γ-producing CD4^+^ and CD8^+^ T cells in HD and KTR patients.

## 2. Material and Methods

### 2.1. Participants and Study Design

In this prospective, observational cohort study, we recruited 40 HD (median age, 72 years; range 43–86; female, 11 [28%]) and 21 KTR patients (median age, 62; range 26–75; female, 7 [33%]). Baseline whole-blood specimens were collected in October 2021, at the time of administration of the booster vaccine dose. The booster dose was administered at a median of 170 days (range: 114–210 days) after full vaccination (two doses) with Spikevax^®^ (Moderna) (n = 46) or Comirnaty^®^ (Pfizer-BioNTech) (n = 15) COVID-19 vaccine, as per local public health authority prescription. Follow-up whole-blood specimens were drawn at a median of 46 days (range 28–81) after receipt of the homologous 3D. In KTR, 3D was administered at a median of 30 months after transplantation.

Participants were enrolled at the Nephrology Service of Hospital Clínico Universitario of Valencia, the Nephrology Service of Hospital Universitario Dr Peset, Valencia, and two satellite hemodialysis centers in Valencia (Spain). Exclusion criteria were current infection, neoplasia, or immunosuppressive treatment (except for KTR) at the time of immunological assessment, and receipt of a solid organ allograft other than the kidney. Patients in the current study had been included in a previous study [24], in which the control group was recruited from among patients’ partners, siblings or household members. Individuals in the control group could not be examined in the current study due to non-eligibility for a booster dose at the time of its initiation. Table 1 summarizes the most relevant clinical characteristics of participants in the two study groups. The study was approved by the Ethics Committee of Hospital Clínico Universitario de Valencia-INCLIVA (2021/194). Informed consent was obtained from all participants.

### 2.2. Immunological Testing

Total (IgG, IgM, and IgA) antiSARS-CoV-2 antibodies were quantified by electrochemiluminescence sandwich immunoassay (ECLIA) Roche Elecsys^®^ Anti-SARS-CoV-2 S (Roche Diagnostics, Pleasanton, CA, USA), and run on a cobas^®^ e601 modular analyzer (Roche Diagnostics, Rotkreuz, Switzerland). The assay is calibrated with the first WHO International Standard and Reference Panel for anti-SARS-CoV-2 antibody [25]. Antibody levels are reported in binding antibody units (BAU)/mL throughout the study. Plasma containing antibody levels above the upper limit of the quantitation (250 BAU/mL) were further diluted and retested. According to the manufacturer, the limit of detection (LOD) of the assay is 0.40 BAU/mL, and responders were those displaying any antibody level above this limit. Previous SARS-CoV-2 infection status of participants was determined by analyzing SARS-CoV-2-nucleocapsid (N)-IgG in plasma (Roche Elecsys^®^ Anti-SARS-CoV-2 IgG (Roche Diagnostics, Pleasanton, CA, USA). 

Neutralizing antibodies (NtAb) targeting the S protein were measured using a GFP-expressing vesicular stomatitis virus pseudotyped with the Wuhan-Hu-1 G614, Delta, and Omicron SARS-CoV-2 variants, as previously described [26]. The LOD of the assay is 20 reciprocal IC50 titer. For anti-RBD antibodies, responders were those who had NtAb titers above the LOD. 

The enumeration of SARS-CoV-2-S-reactive IFN-γ-producing-CD8^+^ and CD4^+^ T cells in fresh whole blood was carried out by flow cytometry for intracellular cytokine staining (BD Fastimmune, Becton Dickinson and Company-Biosciences, San Jose, CA, USA), as previously described [27,28]. For stimulation, we used two sets of 15-mer overlapping peptides (11-mer overlap) encompassing the SARS-CoV-2 Spike (S) glycoprotein (S1, 158 peptides and S2, 157 peptides) at a concentration of 1 μg/mL per peptide (JPT Peptide Technologies GmbH; Berlin, Germany), in the presence of 1  μg/mL of costimulatory monoclonal antibodies (mAbs) to CD28 and CD49d. 

Data are expressed as the number of SARS-CoV-2-reactive IFN-γ-producing CD4^+^ or CD8^+^ T cells relative to the absolute number of CD4^+^ and CD8^+^ T cells, respectively, ×100 (%). Any frequency value of SARS-CoV-2-reactive IFN-γ-producing CD4^+^ or CD8^+^ T cells after background subtraction was considered as a positive (detectable) result and was used for analysis purposes. Anti-RBD antibody and T-cell assays were run at the Microbiology Service of the Hospital Clínico Universitario de Valencia (HCU). NtAb were measured at the Institute for Integrative Systems Biology (I2SysBio), Universitat de Valencia-CSIC.

### 2.3. Statistical Methods

Frequency comparisons for categorical variables were carried out using the Fisher exact test. Differences between medians were compared using the Mann–Whitney U-test, the Wilcoxon test, or the Kruskal–Wallis H test, as appropriate. Two-sided exact *p*-values were reported. A *p*-value < 0.05 was considered statistically significant. The analyses were performed using SPSS version 20.0 (SPSS, Chicago, IL, USA).

## 3. Results

The results shown below should be interpreted, taking the following into account: (i) Four participants were SARS-CoV-2-experienced (all HD) at baseline, as inferred by the presence of detectable anti-SARS-CoV-2-N Ig Gs and historical records of a positive RT-PCR test in upper respiratory tract specimens. (ii) No SARS-CoV-2 infection was documented in any patient between sampling times. (iii) Spikevax^®^ was used for all KTR and 25 HD patients. The remaining 15 HD patients received the Comirnaty^®^ COVID-19 vaccine. (iv) In KTR, 3D was administered at a median of 30 months after transplantation. 

### 3.1. SARS-CoV-2-S Antibody Responses in Participants

**Figure 1:**
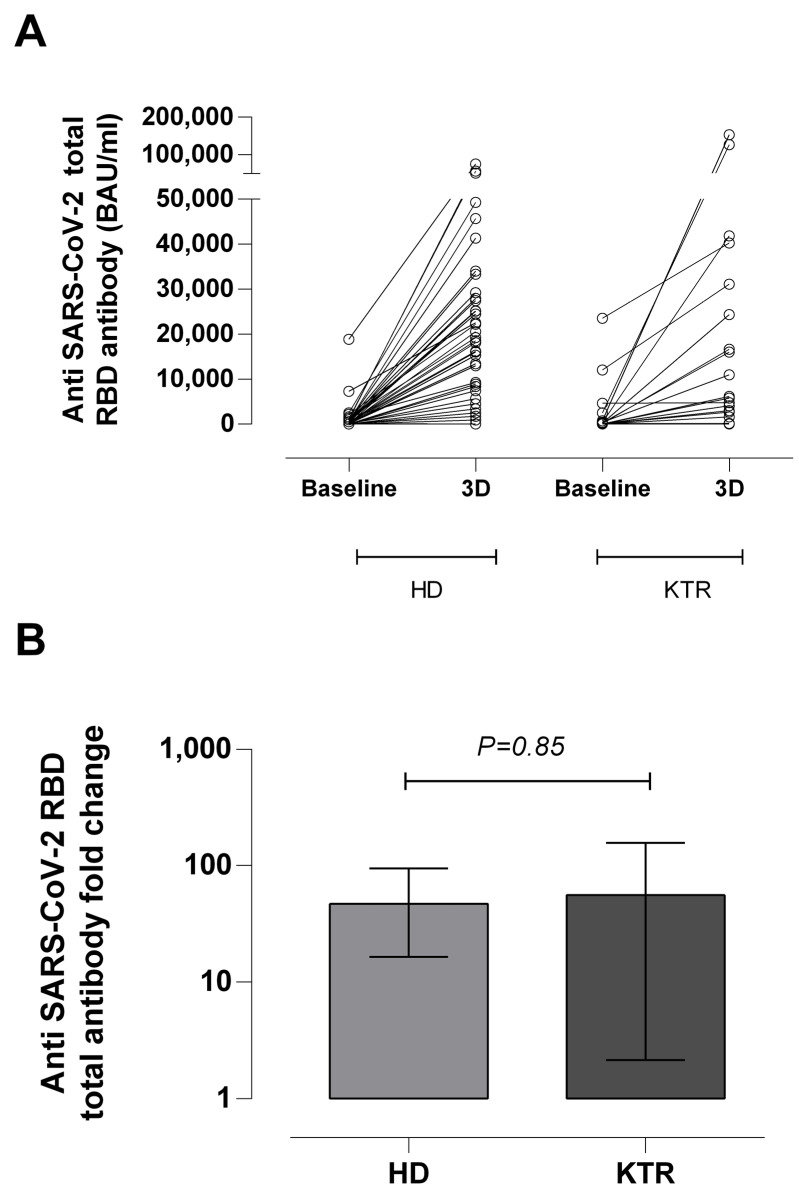
Receptor binding domain (RBD)-reactive total antibodies (in BAU/mL) in hemodialysis patients (HD) and kidney transplant recipients (KTR) after receipt of a homologous mRNA COVID-19 third dose (3D). (**A**) Baseline and 3D antibody levels of participants are shown individually. (**B**) Box and whisker plot depicting the fold-change in anti-RBD antibody levels following 3D in HD and KTR patients. The *p*-value for comparison across groups is shown.

Overall, 39 (98%) of the 40 HD patients had detectable anti-RBD antibodies at baseline (median, 225 BAU/mL; IQR 89–703) and follow-up (median, 18,103 BAU/mL; IQR 7902–27,680), respectively. Only one patient failed to display detectable anti-RBD antibodies after 3D. The receipt of 3D boosted anti-RBD antibody levels, which displayed a median 57-fold increase (IQR 21–118) [Figure 1A,B]. This increase was significantly greater in the n = 36 naïve than in the n = 4 experienced patients (median fold change of 59 vs. median fold change of 12). 

**Figure 2:**
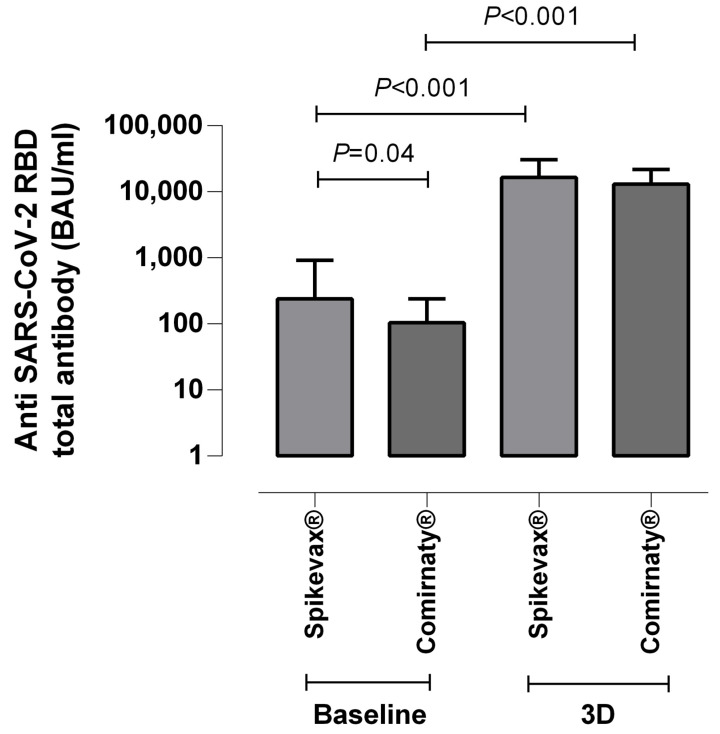
Dot-plot with median and interquartile range depicting receptor binding domain (RBD)-reactive total antibodies (in BAU/mL) measured at baseline and after receipt of a homologous mRNA COVID-19 third dose in hemodialysis patients vaccinated with either Spikevax^®^ or Comirnaty^®^ COVID-19 vaccine; *p*-values for comparisons across groups are shown.

Interestingly, while baseline anti-RBD antibody levels were significantly higher in HD patients vaccinated with Spikevax^®^ than Comirnaty^®^ (*p* = 0.001), no statistically significant differences were found between antiRBD antibody levels after 3D (*p* = 0.14) [Figure 2].

NtAb levels against SARS-CoV-2 Wuhan-Hu-1 G614, Delta, and Omicron variants were measured in 13 HD patients (all naïve), vaccinated with Spikevax^®^ (n = 8), or Comirnaty^®^ (n = 5). The percentage of HD patients displaying detectable NtAb at baseline was 46% (n = 6), 38% (n = 5), and 8% (n = 1) for Wuhan-Hu-1, Delta, and Omicron variants, respectively. The median titers (reciprocal IC_50_) were 127 [IQR 93–150], 47 [IQR 41–52], and 38 (n = 1), respectively. The percentage of responders after 3D increased to 100% (n = 13), 92% (n = 12), and 77% (n = 10), with median NtAB titers of 4620 [IQR 1579–9194], 618 [IQR 451–2172], and 171 [IQR 97–407] for the Wuhan-Hu-1, Delta, and Omicron variants, respectively. All patients showed an increase in NtAB titers against Wuhan-Hu-1, all but one against Delta, and all but three against Omicron. 

**Figure 3:**
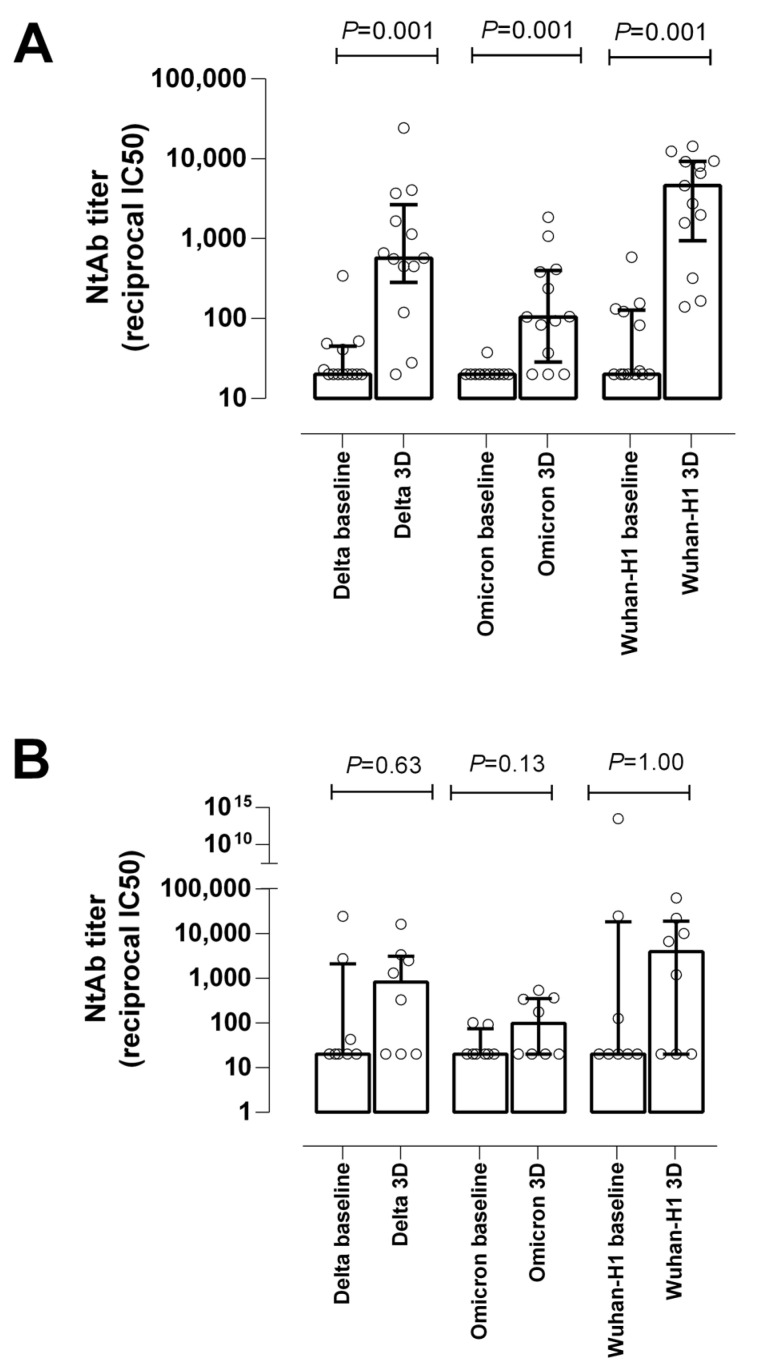
Box and whisker plot depicting the neutralizing antibody (NtAb) levels (reciprocal IC_50_ titer) against the ancestral Wuhan-Hu-1, Delta, and Omicron variants in (**A**) hemodialysis patients (HD) and (**B**) kidney transplant recipients after receipt of a homologous mRNA COVID-19 third dose (3D) relative to those measured at baseline; *p*-values for comparisons across groups are shown.

Overall, the median fold change in NtAb titers was 75 [IQR 25–138], 28 [IQR 13–50], and 5 [IQR 1.9–19] for the respective variants. The increase in NtAb titers was thus more marked against Wuhan-Hu-1 than Delta, and particularly against the Omicron variant (*p* < 0.001) [Figure 3].

Finally, NtAb titers following 3D were not significantly different across HD patients receiving a booster shot of either Spikevax^®^ or Comirnaty^®^ COVID-19 vaccine (*p* = 0.73). Two out of the three patients without NtAB titers against Omicron received Spikevax^®^, and the other received Comirnaty^®^.

Regarding KTR, 17 (80.9%) and 19 (90.5%) of 21 patients shwoed detectable anti-RBD antibodies at baseline (median, 206 BAU/mL; IQR 59–749) and after 3D (median, 10,950 BAU/mL; IQR 4099–31,108), respectively. Therefore, in two out of four non-responders, seroconversion could not be documented at baseline. Antibody levels displayed a median 58-fold increase (IQR 27–157) following receipt of 3D (Figure 1B).

NtAb levels against the SARS-CoV-2 Wuhan-Hu-1 G614, Delta, and Omicron variants were measured in eight KTR (all naïve) patients. The percentage of KTR patients displaying detectable NtAb at baseline was 38% (n = 3), 38% (n = 3), and 25% (n = 2) for the Wuhan-Hu-1, Delta, and Omicron variants, respectively. After 3D, the percentage of responders was 63% (n = 5), 63% (n = 5), and 50% (n = 4), with NtAB titers of 10,037 [IQR 6685–21,858], 2484 [IQR 1304–3346], and 349 [IQR 297–405] for the Wuhan-Hu-1, Delta, and Omicron variants, respectively. Three KTR failed to develop NtAb against all variants tested: one had undetectable anti-RBD antibodies, and the other two had 27 and 131 BAU/mL. The remaining five patients experienced a boost in NtAB titers after 3D. The median fold change was 56 [IQR 40–170], 37 [IQR 13–43], and 9 [IQR 1.6–9.3] for Wuhan-Hu-1, Delta, and Omicron, respectively. Again, as shown in Figure 3B, the increase in NtAb titers was more marked against the Wuhan-Hu-1 than the Delta or Omicron variants.

### 3.2. SARS-CoV-2-S T-Cell Responses in Participants

SARS-CoV-2 S-reactive IFN-T-cell responses in paired whole blood baseline and post-3D specimens were measured in 48 participants (HD, n = 32 and KTR, n = 16). Among HD patients, 28 (88%) had detectable SARS-CoV-2-S-reactive IFN-γ-producing CD8^+^ T cells (median, 0.10%; IQR 0.02–0.51%), 17 (53%) had detectable CD4^+^ T cells (median, 0.11%; IQR 0.05–0.22%), and in 17 (53%), both T-cell subsets were detectable at baseline. The number of HD patients with measurable responses at 3D increased to 31 (97%) for CD8^+^ T cells (median, 0.19%; IQR 0.07–0.71%), 31 (97%) for CD4^+^ T cells (median, 0.54%; IQR 0.21–0.88%), and 31 (97%) for both T-cell subsets. One patient with detectable CD8^+^ T-cell responses at baseline had undetectable CD8^+^ T-cell levels after 3D. In contrast, all patients with detectable CD4^+^ T cells at baseline retained them after 3D.

**Figure 4:**
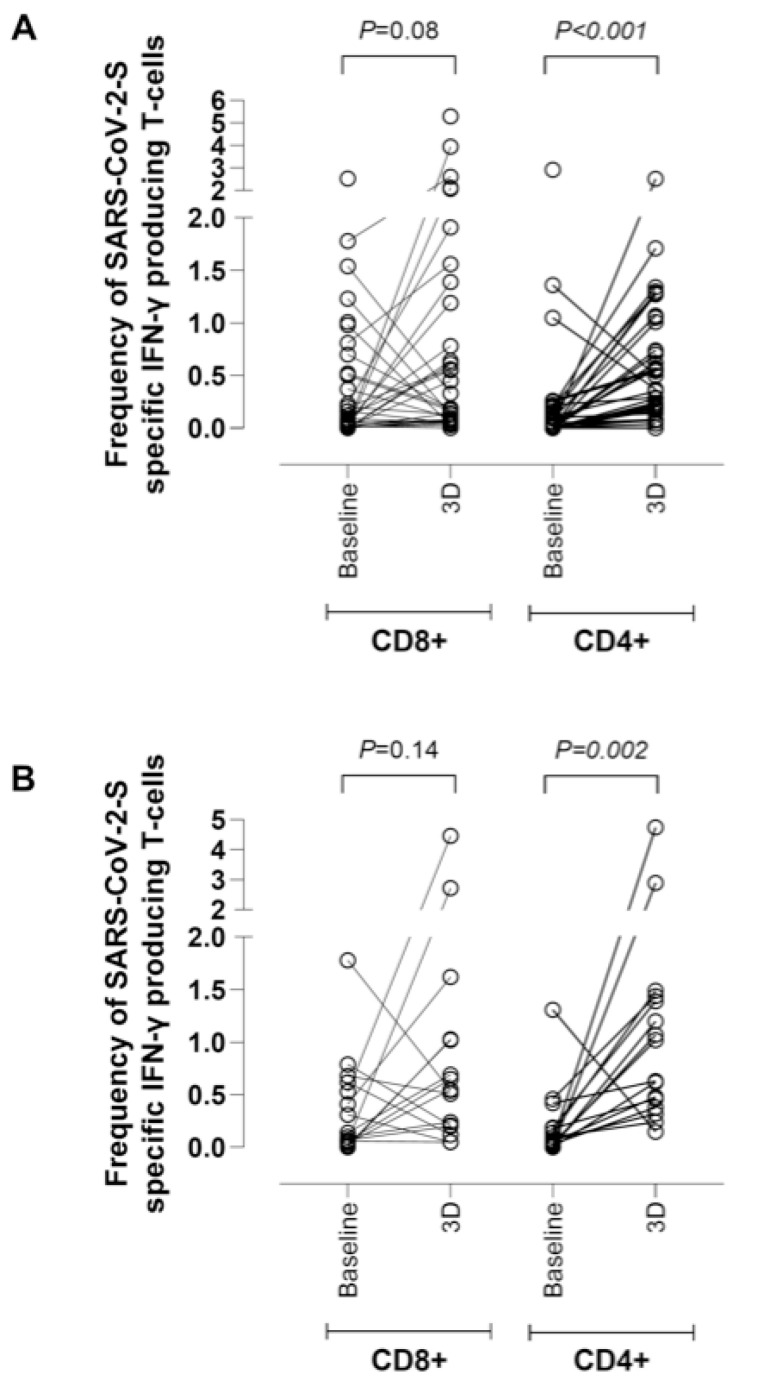
Frequencies of SARS-CoV-2-S- IFN-γ-producing T cells in whole blood as measured by a flow cytometry assay for intracellular cytokine staining in hemodialysis patients (**A**) or kidney transplant recipients (**B**) after receipt of a homologous mRNA COVID-19 third dose (3D) relative to those measured at baseline; *p*-values for comparisons across groups are shown.

As shown in Figure 4, SARS-CoV-2 -S-IFN-γ-producing CD8^+^ T-cell levels increased in 20 (63%) patients following 3D (median difference of 0.53%; IQR 0.07–0.90%). 

**Figure 5:**
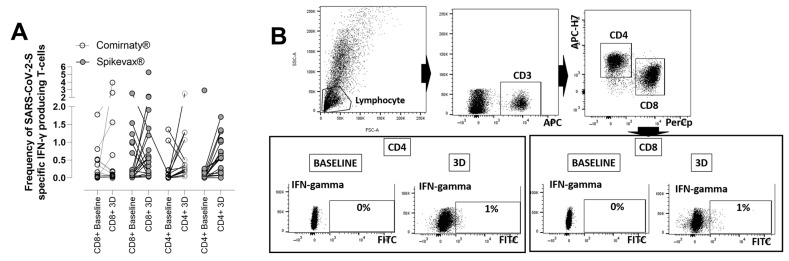
(**A**) Frequencies of SARS-CoV-2-S- IFN-γ producing T cells in whole blood in hemodialysis patients after receipt of an homologous mRNA COVID-19 third dose (3D), either Comirnaty^®^ or Spikevax^®^, relative to those measured at baseline (**B**). Example of an intracellular cytokine staining (ICS) dot plot showing CD4 and CD8 frequencies at baseline and after receipt of a third dose.

SARS-CoV-2 CD4^+^ T-cell levels rose in 29 patients [91%] (median difference of 0.30%; IQR 0.19–0.98%). The patient with undetectable anti-RBD and NtAb responses after 3D had detectable CD8^+^ (2.61%) and CD4^+^ (0.50%) T-cell responses. No differences were found in CD4^+^ and CD8^+^ T-cell responses after 3D across HD patients receiving either Spikevax^®^ or Comirnaty^®^ COVID-19 vaccines (Figure 5).

As shown in Figure 4, SARS-CoV-2 CD8^+^ T-cell levels increased in 10 KTR (63%) following 3D (median difference of 0.79%; IQR 0.52–1.17%), while SARS-CoV-2 CD4^+^ T-cell levels increased in 15 patients [94%] (median difference of 0.98%; IQR 0.31–1.20%).

## 4. Discussion

Herein, we assessed the effect of a homologous SARS-CoV-2 mRNA vaccine booster shot on anti-SARS-CoV-2-S-binding functional antibody and T-cell blood levels in HD and KTR patients. To our knowledge, no comprehensive study has been published to date evaluating both arms of the adaptive immune response after 3D, including NtAb levels against the Omicron variant. In this regard, the difficulty of comparing data gathered by studies using different methods for quantitating anti-S antibodies (chemiluminescent immunoassays with different LODs) or NtAb (wild type virus vs. SARS-CoV-2-S-pseudotyped viruses vs. SARS-CoV-2 surrogate virus neutralization tests) must be underscored. 

Regarding T-cell immunity analyses, it is also worth noting the variety of platforms that have been used for measuring SARS-CoV-2-S-T-cell responses following COVID-19 vaccination in either whole blood or isolated peripheral blood mononuclear cells, including flow cytometry methods for intracellular cytokine (IFN-γ) staining (ICS), such as in the current study, flow cytometry for activation-induced markers (AIM), ELISpot, and IFN-γ-release assays (IGRAs), such as the SARS-CoV-2 Quantiferon^®^ test (QF). Results returned by these assays do not necessarily correlate, either qualitatively or quantitatively [29]. Moreover, differences in the timing of specimen collection after 3D, patient baseline clinical conditions, and SARS-CoV-2 infection status prior to the receipt of 3D further complicate the direct comparison of results across studies.

With regard to the cohort, it is noteworthy that most HD participants in the current study were SARS-CoV-2-naïve prior to 3D, and none were under immunosuppressive therapy at the time of sampling. All but one KTR patients was SARS-CoV-2-naïve, and all had been allografted at least 14 months after receipt of the regular vaccine dose schedule and were thus under maintenance immunosuppression. 

Our data regarding anti-SARS-CoV-2-S antibody responses (anti-RBD, specifically), as measured by a commercially available electrochemiluminescent assay, mostly concurred with previously published data in studies including HD patients receiving either homologous or heterologous mRNA COVID-19 vaccine 3D schedules, and were quantified using similar immunoassays, showing a remarkable booster effect. In summary, all but one (98%) HD patients showed detectable anti-RBD total antibodies after 3D, and their levels were overall notably increased relative to those measured after a two-dose vaccine schedule (median: 57-fold increase). The percentage of detectable anti-SARS-CoV-2-S antibodies in HD at least 15 days after 3D is reported as greater than 85% in most studies, with non-responders being under immunosuppressive therapy [5,6,7,8,9,10]. These studies also report a variable, though uniformly significant increase in anti-S antibody levels [5,6,7,8,9,10]. Interestingly, following 3D, anti-RBD levels were comparable among HD patients receiving Spikevax^®^ or Comirnaty^®^.

In our HD cohort, administering a homologous 3D significantly increased the number of patients displaying detectable NtAb responses against all SARS-CoV-2 variants tested, as measured by a neutralization S-pseudotyped virus assay. However, 3 out of 13 patients evaluated failed to develop NtAb binding the Omicron variant (2 vaccinated with Spikevax^®^ and 1 with Comirnaty^®^). As expected, the booster effect was quantitatively less pronounced against Omicron than the ancestral and Delta variants. In line with our findings, an increase in NtAb levels against the Wuhan-Hu-1 SARS-CoV-2 variant, as determined by a commercially available SARS-CoV-2 surrogate virus neutralization test, has been previously shown in HD patients after a homologous or heterologous vaccine booster shot with Spikevax^®^ or Comirnaty^®^ [8,9].

Regarding KTR patients, 90.5% of participants exhibited detectable anti-RBD antibodies after 3D, a slightly lower percentage than was observed in HD patients. Note that half of the KTR patients with undetectable baseline levels of anti-RBD total antibodies became responders following 3D, in line with previous observations made in KTR patients receiving regular mRNA COVID-19 vaccine schedules, coupled with either homologous or heterologous 3D [14,15,16,17,18,19,20]. Likewise, 3D resulted in an increase in anti-RBD antibody levels of comparable magnitude in KTR and HD patients, although lower values were achieved. A 3D-related booster effect on anti-S-antibody concentrations has been uniformly shown in the abovementioned studies [11,14,15,16,17,18,19,20]. The number of KTR patients with detectable NtAb levels against all variants tested roughly doubled after 3D in the current cohort, in line with previous findings [11,12,17,19,20,22,23]. In addition, an increase in NtAb titers following 3D was registered, regardless of the SARS-CoV-2 variant considered. It is important to note, however, that one-third of participants failed to elicit NtAb against Wuhan-Hu-1, one-third against Delta, and half against Omicron. Among responders, the increase in NtAb titers was notably lower for Omicron than for the rest of the variants tested.

As for HD patients, SARS-CoV-2-S-IFN-γ-producing CD4^+^ or CD8^+^ T-cell immunity was boosted after 3D; increases were observed not only in the percentage of responders, but also in the respective frequencies of both CD8^+^ and CD4^+^ T-cell subsets in whole blood. In fact, only one patient failed to develop SARS-CoV-2-S T cells following 3D. Moreover, the patient with undetectable anti-RBD and NtAb levels was nonetheless able to mount both S-reactive CD4^+^ and CD8^+^ T-cell responses. Importantly, the booster effect of 3D on T-cell immunity was comparable across those vaccinated with Spikevax^®^ or Comirnaty^®^. Our data contrasts with that published by Espi et al. [5], who reported similar percentages of responders at baseline and after 3D (57% vs. 64%), as determined by QF assay. The lower sensitivity of the QF assay compared to our flow cytometry test as the ICS method may account for this discrepancy [29].

Regarding the KTR patients, a key observation was that all patients exhibited measurable SARS-CoV-2-S-IFN-γ-producing CD4^+^ or CD8^+^ T cells after 3D. Moreover, SARS-CoV-2 CD4^+^ T-cell levels increased significantly compared to baseline in 94% of patients in our cohort. SARS-CoV-2 CD8^+^ T cells were only detected in 63% of patients, with no differences across patients vaccinated with Spikevax^®^ or Comirnaty^®^. Likewise, Bertrand et al. [19] reported that all KTR patients vaccinated with Comirnaty^®^ displayed detectable SARS-CoV-2-S T-cell responses at one month after 3D, as determined by ELISpot. Further proof of the beneficial impact of 3D on the development and/or expansion of SARS-CoV-2-S-reactive T-cell responses is that 9 out 10 KTR patients vaccinated with Comirnaty^®^ who failed to respond to two vaccine doses developed T-cell responses, as measured by flow cytometry for AIM and ICS after 3D [18]. The frequency of S-specific IFN-γ-secreting cells was also reported to increase significantly after 3D, even in patients vaccinated with Comirnaty^®^ who remained seronegative, as determined by ELISpot and QF [20].

The current study has several limitations that deserve comment. First, it has a relatively small sample size, which precluded the study of risk factors potentially associated with the failure to mount adaptive immune responses in KTR patients following 3D. Second, NtAbs and T-cell immunity could only be measured in a subset of participants. Third, we lacked a control group, due to the unavailability of the patients’ partners, siblings, or household members who had served as previous controls [24], but who were not eligible for a booster dose following local vaccination guidelines. A recent study of 108 individuals (59 patients on dialysis and 49 controls) showed that patients on HD had a lower antiRBD response after two doses of mRNA vaccine. Serologic and cellular memory response boosted after a third dose, but only in HD patients who had not suffered infection, not in recovered HD patients [30]. A prospective study with more than 1700 participants (1256 HD patients, 368 KTR patients, 144 healthy controls) revealed similar rates of seroconversion after two doses of mRNA vaccine in HD patients and HC (>95%), while the KTR seroconversion rate was only 42%, with lower frequencies of SARS-CoV-2 reactive CD4+ T helper cells [31]. Karaba et al. compared 47 solid organ transplant recipient responses after three doses to 15 HC after two doses. A third SARS-CoV-2 vaccine dose increased neutralizing antibodies (1.4-fold against Delta) median total anti- spike (1.6-fold), and pseudo-neutralization against the variants of concern (2.5-fold vs. Delta). However, the neutralization activity was significantly lower than that in healthy controls (*p* < 0.001). Of solid organ transplant recipients, 32% had zero detectable nAb against the Delta variant after a third dose compared to 100% of controls after the standard two doses vaccination schedule [11]. Data comparing the KTR to the HC response to a third dose is scarce. Benning et al. assessed the humoral immune response in 49 kidney transplant recipients and 25 age-matched healthy controls after a third dose. All healthy controls were seropositive for antispike S1 IgG, surrogate neutralizing antibodies, and anti-RBD antibodies, whereas only 26/49 (53%) of kidney transplant recipients showed concurrent seropositivity. KTR patients also showed significantly impaired neutralization against SARS-CoV-2 wild-type and the B.1.617.2 (Delta) variant when compared to HC. Neutralization against the B.1.1.529 (Omicron) variants in 30 seroconverted kidney transplant recipients was significantly lower compared to that in the 10 healthy controls [32].

We did not investigate whether the immune response was affected differentially depending on the immunosuppressive drugs used, as we centered our comparison on HD/KTR patients, and the sample size precluded any further analysis. Other studies have shown that the immune response to a third BNT262b2 dose is highly influenced by the intensity of the immunosuppressive regimen. Belatacept-treated patients are the worst responders [33,34], developing no antibodies and no or only few specific T cells. Mycophenolic acid has also been associated with a lower seroconversion rate [15]. In a large cohort of 87 KTR patients studied after a third dose, those on cyclosporine were more likely to develop humoral and cellular responses than those on tacrolimus, regardless of mycophenolate mofetil coadministration [34]. In a study on 65 KTR patients, a lack of response to COVID-19 vaccines was associated with African American race, being on high-dose anti-metabolite therapy, and having lower pre-vaccination CD3, CD4 T-cell, and serum IgM levels. No association was found with age or sex [35].

Finally, patients were examined at a single time point after 3D, meaning that the kinetics of antibody and T-cell responses after 3D were not assessed. Studies addressing this issue are currently ongoing.

## 5. Conclusions

In summary, our data indicated that a homologous mRNA COVID-19 vaccine 3D exerts a booster effect on anti-RBD antibodies, NtAb binding to SARS-CoV-2 ancestral and variants of concern (Delta and Omicron), and SARS-CoV-2-S- IFN-γ-producing T cells in HD patients, although this effect appeared to be of a lower magnitude in KTR patients. The remarkable effect of the 3D in both arms of the adaptive immune system in KTR patients is overshadowed by the lack of detectable NtAb responses against the Omicron variant in half of the participants. This latter observation lends support to administering either an additional Wuhan-Hu-1-based booster dose, or when available, an Omicron-adapted vaccine to KTR patients. 

## Figures and Tables

**Table 1:** Demographic and clinical characteristics of patients in the two groups.

Parameter	Study Group	
	HD (40)	KT (21)
Female (%)	11 (28%)	7 (33%)
Age (years) [median (IQR)]	72 (43–86)	62 (26–75)
**Comorbidities (yes)**	37 (93%)	19 (90%)
Hypertension	32 (80%)	19 (90%)
Diabetes	18 (45%)	7 (33%)
Previous cardiovascular disease	13 (33%)	1 (4.8%)
Chronic liver disease	3 (8%)	0%
Obesity	7 (18%)	2 (10%)
**Primary etiology**		
Nephroangioesclerosis	8 (13%)	0%
Diabetic kidney disease	6 (9.8%)	5 (23.7%)
Chronic interstitial nephritis	3 (4.9%)	1 (4.8%)
Cystic disease	3 (4.9%)	0%
Urologic disease	0%	2 (9.5%)
Primary glomerulonephritis	5 (8.2%)	9 (42.9%)
Unknown	14 (23%)	3 (14.3%)
Other	1 (1.6%)	1 (4.8%)
**Type of vaccine**		
Cominarty^®^	15 (38%)	0%
Spikevax^®^	25 (62%)	21 (100%)
**Type of inmunosupression**		
Prednisone dose mg/day [median (IQR)]		5 (1.87–5)
Mycophenolate mofetil (%)		42.9%
Mycophenolate mofetil dose [mg/day (mean ± DE)]		833.3 ± 176.7
Mycophenolic acid (%)		47.6%
Mycophenolic acid dose [mg/day (mean ± DE)]		558 ± 157.6
Calcineurin inhibitor (%)		95.2%
Calcineurin inhibitor [mg/day (mean ± DE)]		2.86 ± 1.67
**Uremic status**		
Kidney graft glomerular filtration rate CKD-EPI [mL/min 1.73 m^2^ (mean ± DE)]		56.4 ± 16.5
Kt/V (mean ± DE)	1.63 ± 0.17	

## Data Availability

The data presented in this study are available on request from the corresponding author. The data are not publicly available due to privacy and ethical reasons.

## Abbreviations

HD	hemodialysis
KTR	kidney transplant recipients
NtAb	neutralizing antibodies
RBD	receptor binding domain
S	SARS-CoV-2-Spike
3D	third mRNA COVID-19 vaccine dose
ECLIA	electrochemiluminescence sandwich immunoassay
